# Applying concurrent multidisciplinary care to gender affirmation in transgender women: A case series

**DOI:** 10.1002/ccr3.3134

**Published:** 2020-07-29

**Authors:** Adam Lloyd, Sara Brenner, Lydia A. Fein, David Rosow

**Affiliations:** ^1^ Department of Otolaryngology University of Miami Miller School of Medicine Miami FL USA; ^2^ University of Miami Miller School of Medicine Miami FL USA; ^3^ Department of Obstetrics, Gynecology, and Reproductive Sciences University of Miami Miller School of Medicine Miami FL USA

**Keywords:** feminizing glottoplasty, gender affirmation, penile inversion vaginoplasty, transgender, voice training

## Abstract

Gender affirmation surgeries, though important for many transgender persons, can be numerous. Combining surgeries, as presented in this case series, affords many benefits to patients without increasing risk or complications.

## Graphical abstract

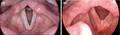

## 1 INTRODUCTION

This case series presents three transgender women who underwent gender‐affirming treatment with concurrent feminizing glottoplasty and penile inversion vaginoplasty. In our series, combining these into one procedure minimized anesthesia exposure and did not increase operative time, hospitalization, or complications, suggesting that they can safely combined.

Gender dysphoria and vocal dysphonia are common among transgender women (TGW).^1^ Gender‐affirming surgical procedures can improve quality of life of transgender persons.^2^ Specifically, voice training and therapy may be effective methods for improving voice perception in transgender women.^3^ However, some may require pitch elevation surgeries to achieve desired pitch. Transgender women also frequently seek genital surgery to construct a neovagina. Despite their benefits, each surgical procedure portends the risks of anesthesia, hospitalization, and complications. As an academic hospital center specializing in comprehensive surgical and therapeutic management of transgender patients, we are often able to combine surgical procedures to minimize these risks. Most recently, we have combined feminizing glottoplasty (FG) and penile inversion vaginoplasty (PIV). This has served to prevent two anesthetics, minimize travel, improve patient care, and decrease cost. We are unaware of previous reports combining these two important gender‐affirming feminizing procedures.

## 2 CASE SERIES

Three patients, age range 45‐51 years old, underwent combined FG and PIV at our center between 2017 and 2018. Each had no prior gender‐affirming surgical procedures. All were nondiabetic, and BMI ranged from 21.6 to 38.1 kg/m^2^. See Table 1 for demographic information.

**Table 1:** Demographic information

Participant	Age	Race	BMI	Medical history	Surgical history	Hormones	Smoker
P1	45	C	38.1	Negative	Left‐hand surgery	Yes	Former smoker
P2	51	C	21.6	Hypertension	Negative	Yes	No
P3	50	C	32.5	Negative	Negative	Yes	Former smoker

Abbreviation: BMI, Body mass index; C, caucasian.

Preoperatively, the patients were seen at our multidisciplinary transgender health center. These patients endorsed gender dysphoria, clinically significant distress, related to both voice and physical appearance and thus were evaluated by both a plastic surgeon and speech‐language pathologist specializing in gender‐affirming care.

Penile inversion vaginoplasty was planned by the plastic surgeon, and all three patients were deemed to be candidates for voice modification therapy. Initial voice evaluation included a measure of voice‐related quality of life with the Transsexual Voice Questionnaire for Male‐to‐Female Transsexuals (TVQMtF) as well as baseline recordings for the patient's current voice and speech characteristics.^4^ Fundamental frequency, the objective correlate of pitch, was collected using the Multidimensional Voice Profile program from Pentax Medical's Computerized Speech Lab (Pentax Medical). Patients were instructed to sustain the sound “ah” in a comfortable speaking pitch for approximately 5 seconds. This task was not modeled so as to not influence habitual pitch production. After six months of voice training without complete satisfaction, they were considered for voice modification surgery, as is offered as an option for increasing pitch.

**Figure 1:**
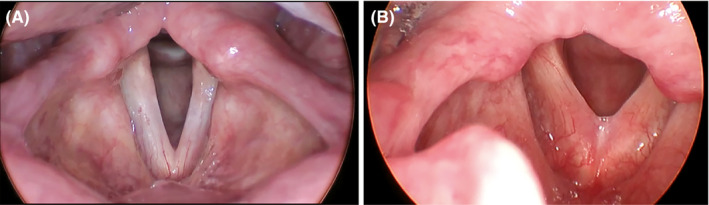
(A) Prefeminization glottoplasty and (B) Postfeminization glottoplasty

Feminizing glottoplasty was performed endoscopically under general anesthesia. The endoscopic approach has been found to have high patient satisfaction rate and ability to increase the fundamental frequency of the voice by creation of an anterior web and fusion reducing vibratory length.^5^ The patients were intubated with a small endotracheal tube, internal diameter of 5.5 mm or less, allowing for easier manipulation of the vocal folds and causing less disturbance of their natural position than a larger tube. Using an operating laryngoscope, a potassium titanyl phosphate (KTP) laser was used to remove the epithelium from both sides of the anterior commissure of the vocal folds, extending back approximately 40% of the glottal length. These denuded edges were then approximated by injection of carboxymethylcellulose gel, a temporary augmentation material, to facilitate web formation between the two sides. Small absorbable endoscopic sutures were placed to further ensure that the two raw surfaces remained in direct contact through the process of scar formation. This permanently shortens the vocal folds by creating an anterior web, reducing the vibratory length, and increasing the fundamental frequency of vibration (Figure 1).

Concurrent to FG, the patients underwent PIV using standard techniques to create a neovagina from inverted penile skin in the fascial space between the bladder and rectum with subsequent tissue rearrangement to create feminine external genitalia.^6^ In this case series, surgical times ranged from 353 to 553 minutes. By comparison, mean surgical time for a cohort of patients undergoing PIV alone at our institution (n = 21) was 457 minutes (SD 114).

As is standard for PIV patients, the patients in this series remained in the hospital for seven‐to‐ten days following surgery. Following FG, patients were required to refrain from using their voice for one week after voice surgery to prevent postoperative trauma and dehiscence of the fusion. At one‐week postsurgery while still inpatient, the speech‐language pathologist initiated voice rehabilitation to support healing and prevent vocal strain, using a gradual reintroduction of voice in a prescriptive manner. Vocal exercises such as resonant voice and semi‐occluded vocal tract exercises were taught to promote strain‐free production and support healing of the vocal folds.^5^, ^7^

No complications arose specific to FG. One patient did experience postoperative venous thromboembolism. All followed up for continued voice and communication therapy after discharge and had post‐PIV follow‐up with plastic surgery. At three‐month follow‐up, all patients had patent, functional, well‐healed neovaginas, increased mean fundamental frequency, and decreased TVQMtF scores (Table 2).

**Table 2:** Vocal outcomes

Patient	Age	Pre TVQMtF	Post TVQMtF	Pre mean fundamental frequency (Hz)	Post mean fundamental frequency (Hz)
P1	45	105/120	49/120	147	166
P2	51	95/120	41/120	134	162
P3	50	78/120	41/120	119	152

Abbreviation: TVQMtF, Transsexual Voice Questionnaire for Male‐to‐Female Transsexuals.

## 3 DISCUSSION

This case series describes our multidisciplinary approach to evaluation and treatment of transgender women seeking gender confirmation. We preliminarily showed that combining PIV and FG is safe, effective, and does not add significantly to operative time or hospital stay. It also affords the benefits of fewer operative visits and exposures to general anesthesia as well as reduces travel and recovery periods for patients, which can lower cost and minimize time away from work. The combined approach allows for voice therapy to be initiated in‐person in the hospital and for adherence to strict voice restriction guidelines in the immediate postoperative period. Based on our findings, there are potential benefits to this combined approach.

In the appropriate setting in which multidisciplinary care can be offered, such as our academic hospital‐based gender affirmation program, PIV and FG can be safely and effectively combined. Further investigation to evaluate long‐term outcomes and results in a larger cohort of patients is warranted, though great promise is shown based on the patients in this series.

## CONFLICT OF INTEREST

The authors report no conflicts of interest.

## AUTHOR CONTRIBUTION

AL, LAF, and DR: collected data; developed the manuscript. SB: developed the manuscript.
